# Vitamin D Deficiency and Outcome of COVID-19 Patients

**DOI:** 10.3390/nu12092757

**Published:** 2020-09-10

**Authors:** Aleksandar Radujkovic, Theresa Hippchen, Shilpa Tiwari-Heckler, Saida Dreher, Monica Boxberger, Uta Merle

**Affiliations:** 1 Department of Internal Medicine V, University of Heidelberg, 69121 Heidelberg, Germany; aleksandar.radujkovic@med.uni-heidelberg.de; 2 Department of Internal Medicine IV, University of Heidelberg, 69121 Heidelberg, Germany; theresa.hippchen@med.uni-heidelberg.de (T.H.); shilpa.tiwari-heckler@med.uni-heidelberg.de (S.T.-H.); saida.dreher@med.uni-heidelberg.de (S.D.); monica.boxberger@med.uni-heidelberg.de (M.B.)

**Keywords:** vitamin D, SARS-CoV-2, COVID-19, outcome, severity, retrospective

## Abstract

Infection with the severe acute respiratory syndrome coronavirus-2 (SARS-CoV-2) poses an enormous challenge to health care systems throughout the world. Without causal treatment, identification of modifiable prognostic factors may help to improve outcomes. To explore possible associations of vitamin D (VitD) status with disease severity and survival, we studied 185 patients diagnosed with coronavirus disease 2019 (COVID-19) and treated at our center. VitD status at first presentation was assessed retrospectively using accredited laboratory methods. VitD deficiency was defined as serum total 25-hydroxyvitamin D level < 12 ng/mL (<30 nM). Primary endpoint was severe course of disease (i.e., need for invasive mechanical ventilation and/or death, IMV/D). Within a median observation period of 66 days (range 2–92), 23 patients required IMV. A total of 28 patients had IMV/D, including 16 deaths. Ninety-three (50%) patients required hospitalization (inpatient subgroup). A total of 41 (22%) patients were VitD deficient. When adjusted for age, gender, and comorbidities, VitD deficiency was associated with higher risk of IMV/D and death (HR 6.12, 95% CI 2.79–13.42, *p* < 0.001 and HR 14.73, 95% CI 4.16–52.19, *p* < 0.001, respectively). Similar correlations were observed in the inpatient subgroup. Our study demonstrates an association between VitD deficiency and severity/mortality of COVID-19, highlighting the need for interventional studies on VitD supplementation in SARS-CoV-2 infected individuals.

## 1. Introduction

Infection with the viral pathogen severe acute respiratory syndrome coronavirus-2 (SARS-CoV-2) has reached pandemic status in 2020. With more than 700,000 deaths attributed to coronavirus disease 2019 (COVID-19) at the time of writing [1], COVID-19 poses an enormous challenge to societies and health care systems throughout the world.

Clinical features of COVID-19 may vary from asymptomatic or mild upper respiratory tract symptoms to a severe acute lung injury with subsequent systemic inflammation, multiorgan failure, and fatal outcome. Currently, there is no causal treatment for COVID-19. Advanced age, male gender, and underlying comorbidities were shown to be associated with severe COVID-19 [2,3,4]. However, none of these risk factors are modifiable and little is known about the potential protective determinants.

The active form of vitamin D3, 1α, 25-dihydroxyvitamin D3 (1,25D3), also known as calcitriol, is a pluripotent hormone and important modulator of both innate and adaptive immunity [5], and serum total 25-hydroxyvitamin D (25(OH)D) is commonly used to assess individual vitamin D (VitD) status [6]. Low VitD status was shown to be associated with various clinical conditions including increased susceptibility to infectious disease, but its causal role remains controversial.

Notably, in their large meta-analysis of 25 randomized controlled trials, Martineau et al. [7] demonstrated that VitD supplementation protects against acute respiratory tract infections, particularly in patients presenting with very low VitD status (25(OH)D < 10 ng/mL). Their study has received renewed attention recently, leading to a lively discussion on the potential impact of VitD status on mortality from SARS-CoV-2 infection and on VitD supplementation as a possible therapeutic approach for COVID-19 [5,8,9,10,11,12,13,14].

However, data on VitD status in the context of clinical outcomes of SARS-CoV-2 infection are limited. In the present study, we therefore sought to explore possible associations between VitD status and disease severity and survival in COVID-19 patients.

## 2. Materials and Methods

### 2.1. Patients and Data Collection

Consecutive symptomatic SARS-CoV-2-positive patients admitted to the Medical University Hospital Heidelberg were enrolled onto a prospective non-interventional register. Included in the analysis were patients diagnosed and treated between 18 March and 18 June 2020 who had consented to study participation and had serum samples available for analysis. Written informed consent according to the Declaration of Helsinki was obtained for all patients and the local ethics committees had approved data collection and analysis (reference number: S-148/2020). Patient data and follow-up were assessed prospectively except for information on medication at admission which was assessed in retrospect by review of the records.

### 2.2. Diagnosis, Supportive Care and Treatment

Patients were tested for SARS-CoV-2 infection following local guidelines and in accordance with the latest recommendations of the Robert Koch Institute [15]. For diagnosis of SARS-CoV-2, RNA was isolated from nasopharyngeal and oropharyngeal swab specimens using QIAGEN Kits (QIAGEN, Hilden, Germany), automated on the QIASymphony (DSP Virus/Pathogen Mini Kits) or QIAcube (QIAamp Viral RNA Mini Kits) devices, and eluted in 115 μL elution buffer. RT-PCR was carried out using various reagent mixes—LightMix Modular SARS and Wuhan CoV E-gene, LightMix Modular SARS and Wuhan CoV N-gene, LightMix Modular Wuhan CoV RdRP-gene, and LightMix Modular EAV RNA Extraction Control (as internal control) from TIB MOLBIOL Syntheselabor GmbH (Berlin, Germany), and LightCycler Multiplex RNA Virus Master (Roche, Mannheim, Germany)—according to manufacturer’s instructions. RT-PCR was performed on LightCycler 480 or 480 II (Roche, Germany).

The decision for inpatient versus outpatient admission was based on the level of spontaneous oxygen saturation (SpO_2_ ≤ 93%), comorbidities, and the overall performance status. With regard to established COVID-19 severity classifications [16], all inpatients had severe disease (defined as tachypnea [≥30 breaths per min], oxygen saturation ≤ 93% at rest, or PaO_2_/FiO_2_ ratio < 300 mm Hg) or critical disease (respiratory failure requiring mechanical ventilation, septic shock, or other organ dysfunction or failure that requires intensive care).

Outpatients included in the analysis had symptomatic disease presenting with fever, cough, sore throat, myalgia, and/or fatigue. Outpatients were visited in their home quarantine on a regular basis and their clinical conditions were regularly evaluated employing “Coronataxis” (i.e., home visits by medical students, nursing stuff, and a supervising physician) which were implemented by the University Hospital Heidelberg and the regional health authorities [17].

Oxygen therapy included oxygen delivery via nasal cannula, high-low nasal oxygen therapy (HFNO), and invasive mechanical ventilation (IMV). Criteria for initiation of IMV were failure to maintain adequate ventilation or oxygenation in spite of high FiO_2_ delivery.

Hospitalized patients were treated with standard supportive care including antibiotic and antifungal therapy, whereas additional immunomodulatory therapy was inconsistently applied (azithromycin, hydroxychloroquine, tocilizumab, anakinra, prednisolone, maraviroc, Cytosorb™, and plasmapheresis). Routine CT scans were performed at hospital admission for most patients.

### 2.3. Assessment of VitD Status and Cytokine Serum Levels

To assess whole-body VitD status of patients, levels of total 25(OH)D were measured retrospectively in cryopreserved (−80 °C) serum samples collected in gel tubes at the time of admission and SARS-CoV-2 testing. Serum levels of total 25(OH)D were quantified using a commercially available immunoassay (ADVIA Centaur Vitamin D Total Assay^®^, Siemens Healthcare GmbH, Erlangen, Germany).

In inpatients, serum levels of Interleukin-6 (IL-6) were measured prospectively at the time of hospitalization. IL-6 was quantified using a commercially available immunoassay (IMMULITE^®^ Immunoassay System, Siemens Healthcare GmbH, Erlangen, Germany).

All measurements were carried out at the Department of Clinical Chemistry of the Heidelberg University Hospital using accredited laboratory methods (certified according to ISO 15189 by Germany’s national accreditation body).

### 2.4. Statistics

Categorical data of patient characteristics were compared using the Fisher exact test. Continuous variables were compared applying the Mann-Whitney U test. Median follow-up time was calculated by the reverse Kaplan-Meier method [18].

Primary endpoint was severe course of disease (i.e., need for invasive mechanical ventilation and/or death, IMV/D, as a composite endpoint). Secondary endpoint was death of any cause.

Survival was calculated from the date of first presentation/admission and SARS-CoV-2 testing to last follow-up or death of any cause. Patients alive were censored at the date of last contact. Severe course of the disease was determined as time from the date of first presentation/admission and SARS-CoV-2 testing to IMV/D. Patients who were alive without necessary IMV were censored at the time of the last contact.

VitD deficiency was defined as serum total 25(OH)D level < 12 ng/mL (equivalent to <30 nM). In addition, the cut-point of 25(OH)D < 20 ng/mL (<50 nM) reflecting “VitD insufficiency” was analyzed [19].

For uni- and multivariable analysis of the associations between VitD status and severe course of the disease and survival, Cox regression models were applied. For the multivariable analyses, additional prognostic factors including age, gender, and presence of comorbidity were chosen to reflect confounders demonstrated to be associated with risk of death in previous reports [2,3,4].

All statistical tests were two-sided at a significance level of 5%. Hazard ratios (HR) were estimated with 95% confidence interval (95% CI). Calculations were done using IBM^®^ SPSS^®^ Statistics, Version 24.0.0.

## 3. Results

### 3.1. Patients and Treatment Characteristics and VitD Status

Ninety-two (50%) patients were followed in the outpatient setting and 93 (50%) patients required hospitalization. The patient and treatment characteristics of the entire cohort and the inpatient versus outpatient subgroups are given in Table 1. Hospitalized patients were older, were predominantly male, and had substantially more comorbid conditions.

**Figure 1:**
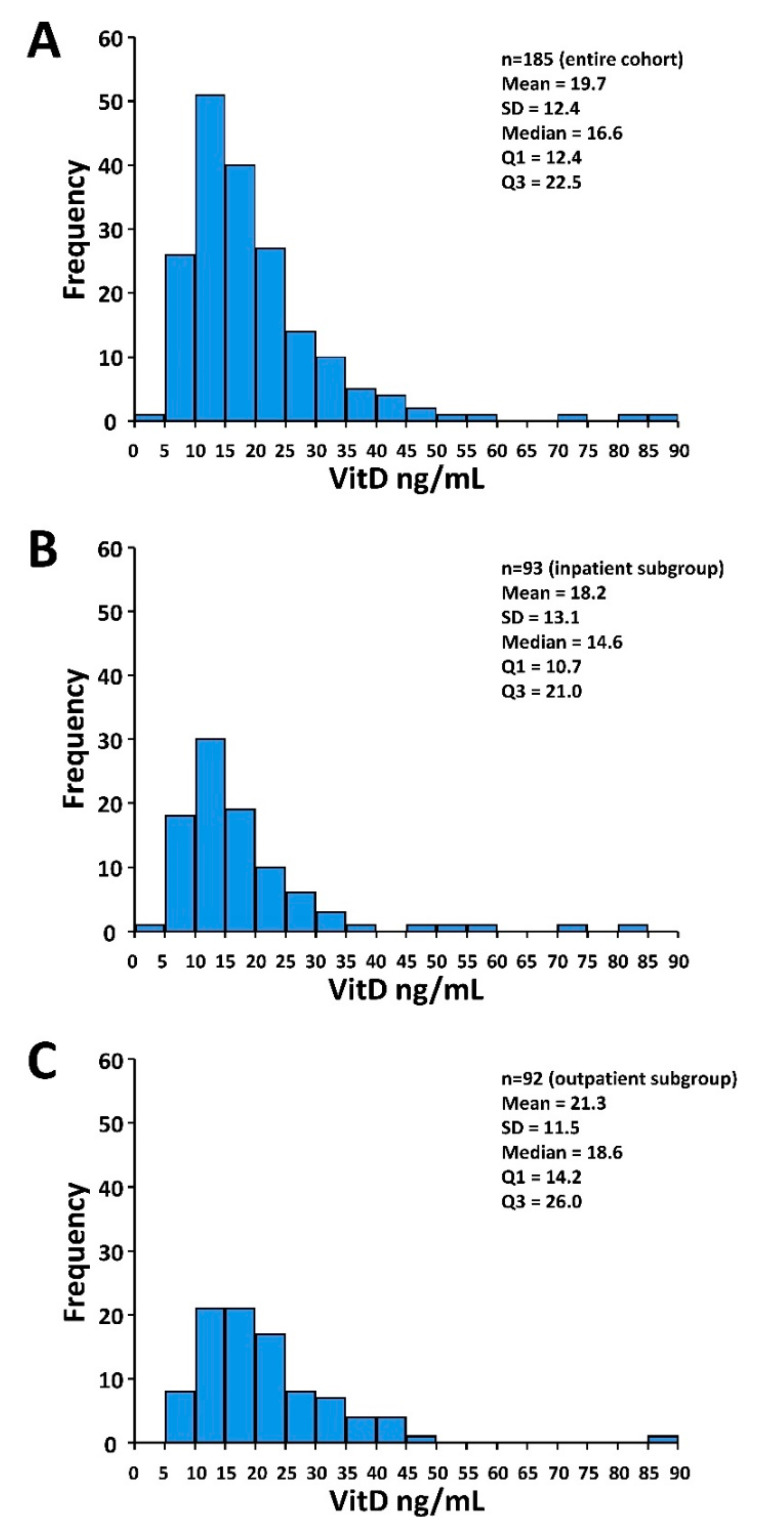
Histograms of the VitD distribution. (**A**) Entire cohort (*n* = 185). (**B**) Inpatient subgroup (*n* = 93). (**C**) Outpatient subgroup (*n* = 92). Abbreviations: Q1 and Q3, first and third quartile, respectively; SD, standard deviation.

For the entire cohort, median VitD level was 16.6 ng/mL (interquartile range 12.4–22.5). A total of 41 (22%) patients were VitD-deficient (<12 ng/mL) and 118 (64%) patients had VitD levels < 20 ng/mL. Median VitD level was significantly lower in the inpatient versus the outpatient subgroup. Accordingly, a higher proportion of inpatients was VitD-deficient (VitD < 12 ng/mL) (Table 1). The distributions of the VitD levels including descriptive statistics for the entire cohort and the subgroups are depicted in Figure 1.

Median follow-up was 66 days (range 2–92 days). A total of 16 patients died and 23 patients required invasive mechanical ventilation. The IMV/D event occurred in 28 patients. All events occurred in hospitalized patients.

### 3.2. Patient and Treatment Characteristics According to VitD Status

The patient and treatment characteristics according to VitD status in the entire cohort and the inpatient and outpatient subgroups are summarized in Table 2. In the entire cohort, among VitD-deficient patients, median age and hospitalization rate was higher and more patients required (intensive) oxygen therapy (Table 2). The findings were similar in the subgroup of hospitalized patients. Notably, in VitD-deficient patients, median IL-6 levels at hospitalization were significantly higher (70.5 versus 29.7 pg/mL, Table 2). In the outpatient subgroup, no differences between VitD-deficient patients and patients with VitD levels ≥ 12 ng/mL were observed.

### 3.3. Associations of VitD Status with the Endpoints Invasive Mechanical Ventilation and/or Death and Death

**Figure 2:**
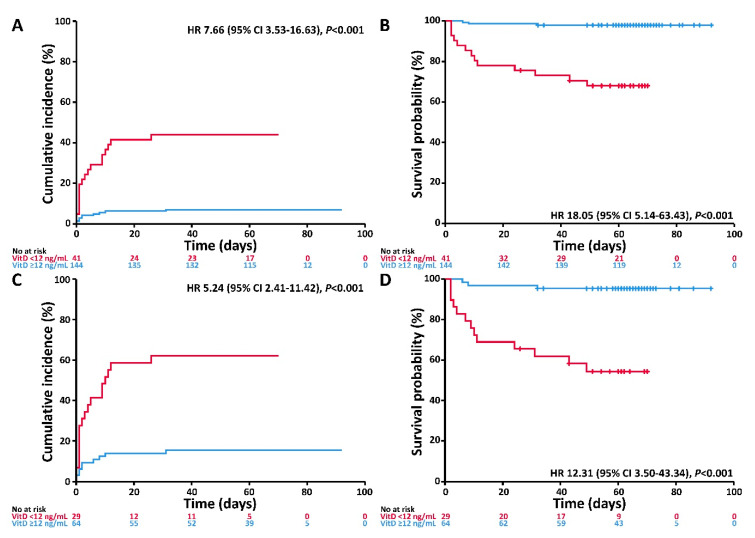
Cumulative incidence of invasive mechanical ventilation and/or death and probability of survival according to VitD status (<12 versus ≥12 ng/mL). (**A**) Cumulative incidence of the composite event invasive mechanical ventilation and/or death according to VitD status in the entire cohort. (**B**) Survival probability according to VitD status in the entire cohort. (**C**) Cumulative incidence of the composite event invasive mechanical ventilation and/or death according to VitD status in the inpatient subgroup. (**D**) Survival probability according to VitD status in the inpatient subgroup. Abbreviations: CI, confidence interval; HR, hazard ratio; VitD, vitamin D.

**Figure A1:**
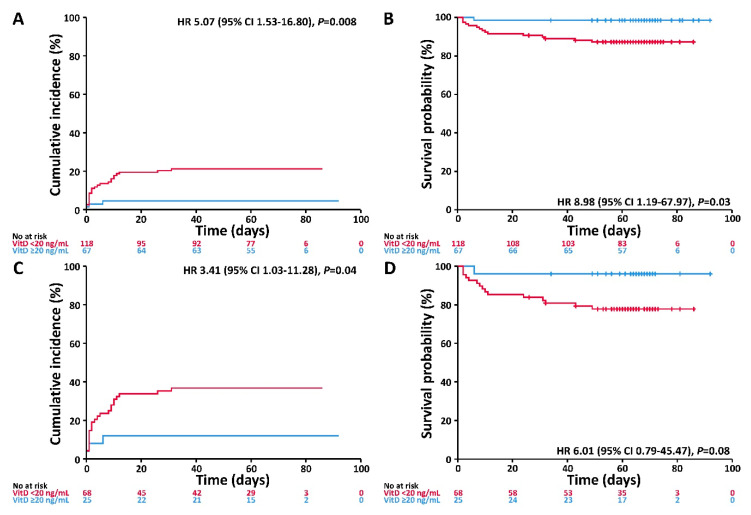
Cumulative incidence of invasive mechanical ventilation and/or death and probability of survival in patients with VitD < 20 versus ≥ 20 ng/mL. (**A**) Cumulative incidence of the composite event invasive mechanical ventilation and/or death according to VitD status in the entire cohort. (**B**) Survival probability according to VitD status in the entire cohort. (**C**) Cumulative incidence of the composite event invasive mechanical ventilation and/or death according to VitD status in the inpatient subgroup. (**D**) Survival probability according to VitD status in the inpatient subgroup.

VitD deficiency at admission was associated with higher incidence of IMV/D and worse survival. The corresponding univariable associations for the entire study cohort are depicted in Figure 2A,B, respectively. Adjusting for age, gender, and comorbidities, VitD deficiency was associated with higher risk of IMV/D and death (HR 6.12 and 14.73, respectively) (Table 3). In the inpatient subgroup, the respective correlations were comparable (Figure 2C,D, Table 3). When applying the VitD cut-off of 20 ng/mL, similar, albeit weaker, associations with incidence of IMV/D and survival were observed (see Table A1 and Figure A1, Appendix A).

## 4. Discussion

The present study demonstrates an association between VitD deficiency and severity of COVID-19. VitD-deficient patients had a higher hospitalization rate and required more (intensive) oxygen therapy and IMV. In our patients, when adjusted for age, gender, and comorbidities, VitD deficiency was associated with a 6-fold higher hazard of severe course of disease and a ~15-fold higher risk of death.

Currently, to the best of our knowledge, there are only a few published studies on VitD in COVID-19 patients. Many observational and prospective studies are still ongoing or initiating and their results are eagerly awaited [20]. With regard to the available literature, D’Avolio et al. showed that 25(OH)D concentrations were lower in patients with positive PCR for SARS-CoV-2 [21], proposing VitD supplementation as a useful measure to reduce the risk of infection. In a recently published meta-analysis, which included mostly patient data from non-peer-reviewed sources, a prognostic relevance of VitD was suggested arguing that diagnosis of VitD deficiency could be a helpful adjunct in assessing patients’ potential of developing severe COVID-19 [22]. Notably, Faul et al. [23] in their study on 33 patients with SARS-CoV-2-related pneumonia reported that VitD deficiency (baseline 25(OH)D < 12 ng/mL) was associated with a significantly increased risk for IMV, which appears in line with our observations.

It should be noted that the cut-off VitD level for determining VitD deficiency or adequacy is subject to debate. Among our patients, 22% and 64% had VitD levels below 12 and 20 ng/mL, respectively, which is in accordance with prevalence estimates reported for adults in Germany [24]. In the present study, VitD deficiency was defined as a serum level of total 25(OH)D < 12 ng/mL consistent with the Institute of Medicine (IOM) position [19]. However, when applying the 20 ng/mL cut-off, which, as per IOM recommendations, is likely to meet the needs of about 97.5% of the general population [19], the associations between low VitD status and severity of COVID-19 were maintained.

We are aware of the limitations of our study, being a single-center, retrospective, and observational study. In particular, since the number of events is rather low, our results require confirmation in larger patient cohorts analyzing a higher number of events and considering additional potential confounders like obesity (as reflected by the body mass index) or other specific comorbidities. Furthermore, it should also be noted that without randomized controlled trial evidence, no causal association between VitD deficiency and severity/outcome of COVID-19 can be inferred. However, since no causal treatment for COVID-19 is available, identification of modifiable prognostic factors may help to improve outcomes. Our results corroborate previous reports [22,23] on VitD as a potential determinant of disease severity supporting assessment of VitD status in all SARS-CoV-2 infected individuals.

Micronutrients are essential in orchestrating a wide range of physiological functions to maintain overall health and support the fight against diseases. Therefore, optimal VitD levels should be considered in all individuals. In particular, VitD deficiency is a medically accepted condition that requires treatment. As regards VitD supplementation, doses and risks are established and well quantified, and general recommendations and intake guidance exist [19]. Therefore, in view of our results and the above considerations, and since an individual’s VitD status is easily modifiable, VitD supplementation should probably be considered for all individuals at high risk of potentially fatal COVID-19 outcome. Bearing in mind the results of the large meta-analysis of VitD supplementation for prevention of acute respiratory tract infections [7], daily or weekly supplementation without additional bolus doses is likely to offer the most benefit in this regard.

In summary, this observational study among patients with COVID-19 who have experienced a definite outcome shows an association between VitD status and severity of and mortality from COVID-19. Prospective, randomized controlled studies on VitD supplementation in SARS-CoV-2 infected individuals are highly warranted.

## Figures and Tables

**Table 1:** Patient and treatment characteristics.

	Entire Cohort	Inpatients	Outpatients	*p* ^a^
	*n* = 185	*n* = 93	*n* = 92	
Median Age [years] (IQR)	60 (49–70)	63 (52–74)	56 (42–64)	<0.001
Age, *n* (%)				0.02
<60 years	88 (48)	36 (39)	52 (57)	
≥60 years	97 (52)	57 (61)	40 (43)	
Gender, *n* (%)				0.003
Male	95 (51)	59 (63)	36 (39)	
Female	90 (49)	34 (37)	56 (61)	
Cardiovascular disease ^b^, *n* (%)				<0.001
Yes	58 (31)	45 (48)	13 (14)	
No	127 (69)	48 (52)	79 (86)	
Diabetes, *n* (%)				0.14
Yes	19 (10)	13 (14)	6 (7)	
No	166 (90)	80 (86)	86 (93)	
Chronic kidney disease, *n* (%)				0.006
Yes	8 (4)	8 (9)	0 (0)	
No	177 (96)	85 (91)	92 (100)	
Chronic lung disease, *n* (%)				0.28
Yes	15 (8)	10 (11)	5 (5)	
No	170 (92)	83 (89)	87 (95)	
Active or history of malignancy, *n* (%)				1
Yes	17 (9)	9 (10)	8 (9)	
No	168 (91)	84 (90)	84 (91)	
Comorbidity, *n* (%)				<0.001
Any	77 (42)	52 (56)	25 (27)	
None	108 (58)	41 (44)	67 (73)	
Median VitD level [ng/mL] (IQR)	16.6 (12.4–22.5)	14.6 (10.7–21.0)	18.6 (14.2–26.0)	0.001
Median IL-6 ^c^ [pg/mL] (IQR)	35.8 (14.4–80.0)	35.8 (14.4–80.0)	-	-
VitD, *n* (%)				0.004
<12 ng/mL	41 (22)	29 (31)	12 (13)	
≥12 ng/mL	144 (78)	64 (69)	80 (87)	
Maximum oxygen therapy ^c^, *n* (%)				<0.001
None	105 (57)	13 (14)	92 (100)	
Low-dose oxygen (NC)	45 (24)	45 (48)	0 (0)	
HFNO	12 (6)	12 (13)	0 (0)	
IMV	23 (12)	23 (25)	0 (0)	
Vitamin D supplementation ^d^, *n* (%)				0.36
Yes	6 (5)	6 (6)	0 (0)	
No	108 (95)	87 (94)	21 (100)	
Unknown	71		71	

Abbreviations: HFNO, high-low nasal oxygen therapy; IMV, invasive mechanical ventilation; IQR, interquartile range; NC, nasal cannula; VitD, vitamin D. ^a^ Inpatients versus outpatients. ^b^ Including arterial hypertension. ^c^ Assessed only in inpatients at hospitalization. ^d^ Maximum received oxygen therapy during hospitalization.

**Table 2:** Patient and treatment characteristics according to baseline VitD status.

	Entire Cohort			Inpatient Subgroup			Outpatient Subgroup		
	(*n* = 185)			(*n* = 93)			(*n* = 92)		
	VitD <12 ng/mL	VitD ≥12 ng/mL	*P* ^a^	VitD < 12 ng/mL	VitD ≥ 12 ng/mL	*P* ^a^	VitD < 12 ng/mL	VitD ≥ 12 ng/mL	*p* ^a^
	*n* = 41	*n* = 144		*n* = 29	*n* = 64		*n* = 12	*n* = 80	
Median age [years] (IQR)	66 (53–78)	58 (47–67)	0.002	71 (54–79)	62 (50–70)	0.03	60 (48–77)	55 (42–63)	0.22
Age, *n* (%)			0.12			0.36			0.76
<60 years	15 (37)	73 (51)		9 (31)	27 (42)		6 (50)	46 (58)	
≥60 years	26 (63)	71 (49)		20 (69)	37 (58)		6 (50)	34 (42)	
Gender, *n* (%)			0.6			0.82			0.76
Male	23 (56)	72 (50)		19 (66)	40 (62)		4 (33)	32 (40)	
Female	18 (44)	72 (50)		10 (34)	24 (38)		8 (67)	48 (60)	
Cardiovascular disease ^b^, *n* (%)			0.06			0.26			0.69
Yes	18 (44)	40 (28)		17 (59)	28 (44)		1 (8)	12 (15)	
No	23 (56)	104 (72)		12 (41)	36 (56)		11 (92)	68 (85)	
Diabetes, *n* (%)			0.04			0.33			0.17
Yes	8 (20)	11 (8)		6 (21)	7 (11)		2 (17)	4 (5)	
No	33 (80)	133 (92)		23 (79)	57 (89)		10 (83)	76 (95)	
Chronic kidney disease, *n* (%)			1			1			1
Yes	2 (5)	6 (4)		2 (7)	6 (9)		0 (0)	0 (0)	
No	39 (95)	138 (96)		27 (93)	58 (91)		12 (100)	80 (100)	
Chronic lung disease, *n* (%)			0.1			0.07			0.61
Yes	6 (15)	9 (6)		6 (21)	4 (6)		0 (0)	5 (6)	
No	35 (85)	135 (94)		23 (79)	60 (94)		12 (100)	75 (94)	
Active or history of malignancy, *n* (%)			0.54			0.45			1
Yes	5 (12)	12 (8)		4 (14)	5 (8)		1 (8)	7 (9)	
No	36 (88)	132 (92)		25 (86)	59 (92)		11 (92)	73 (91)	
Comorbidity, *n* (%)			0.11			0.26			1
Any	22 (54)	55 (38)		19 (66)	33 (52)		3 (25)	22 (27)	
None	19 (46)	89 (62)		10 (34)	31 (48)		9 (75)	58 (73)	
Median IL-6 ^c^ [pg/mL] (IQR)	70.5 (32.0–326.3)	29.7 (14.3–59.9)	0.01	70.5 (32.0–326.3)	29.7 (14.3–59.9)	0.01	-	-	-
Treatment mode, *n* (%)			0.004			1			1
Inpatient	29 (71)	64 (44)		29 (100)	64 (100)		0 (0)	0 (0)	
Outpatient	12 (29)	80 (56)		0 (0)	0 (0)		12 (100)	80 (100)	
Maximum oxygen therapy ^d^, *n* (%)			<0.001			0.004			1
None	15 (37)	90 (63)		3 (10)	10 (16)		12 (100)	80 (100)	
Low-dose oxygen (NC)	8 (20)	37 (26)		8 (28)	37 (58)		0 (0)	0 (0)	
HFNO	4 (10)	8 (6)		4 (14)	8 (13)		0 (0)	0 (0)	
IMV	14 (34)	9 (6)		14 (48)	9 (14)		0 (0)	0 (0)	
Vitamin D supplementation ^e^, *n* (%)			0.19			0.17			1
Yes	0 (0)	6 (7)		0 (0)	6 (9)		0 (0)	0 (0)	
No	30 (100)	78 (93)		29 (100)	58 (91)		1 (100)	20 (100)	
Unknown	11	60					11	60	

Abbreviations: HFNO, high-low nasal oxygen therapy; IMV, invasive mechanical ventilation; IQR, interquartile range; NC, nasal cannula; VitD, vitamin D. ^a^ Patients with VitD < 12 ng/mL versus patients with VitD ≥ 12 ng/mL. ^b^ Including arterial hypertension. ^c^ Assessed only in inpatients at hospitalization. ^d^ Maximum received oxygen therapy during hospitalization. ^e^ Prior to and at admission.

**Table 3:** Multivariable analysis of the effect of VitD deficiency on the endpoints invasive mechanical ventilation and/or death and death in the entire cohort and in the inpatient subgroup.

		Entire Cohort (*n* = 185)				Inpatient Subgroup (*n* = 93)			
		IMV/D (Events, *n* = 28)		Death (Events, *n* = 16)		IMV/D (Events, *n* = 28)		Death (Events, *n* = 16)	
		HR (95% CI)	*p*	HR (95% CI)	*p*	HR (95% CI)	*p*	HR (95% CI)	*p*
Covariate	Effect								
VitD	<12 ng/mL	6.12 (2.79–13.42)	<0.001	14.73 (4.16–52.19)	<0.001	4.65 (2.11–10.25)	<0.001	11.51 (3.24–40.92)	<0.001
Age	≥60 years	3.20 (1.05–9.71)	0.04	7.70 (0.99–58.81)	0.05	3.40 (1.11–10.41)	0.03	8.71 (1.11–68.18)	0.04
Gender	Male	1.69 (0.76–3.76)	0.20	2.50 (0.80–7.81)	0.12	1.40 (0.62–3.19)	0.41	2.15 (0.68–6.78)	0.19
Comorbidity	Any	2.70 (1.04–7.04)	0.04	5.30 (0.96–19.27)	0.06	1.55 (0.59–4.09)	0.37	2.60 (0.58–11.73)	0.21

Abbreviations: CI confidence interval; HR, hazard ratio; IMV/D, invasive mechanical ventilation and/or death; VitD, vitamin D.

## Appendix A

**Table A1:** Multivariable analysis of the effect of VitD insufficiency (VitD < 20 ng/mL) on the endpoints invasive mechanical ventilation and/or death and death in the entire cohort and in the inpatient subgroup.

		Entire Cohort (*n* = 185)				Inpatient Subgroup (*n* = 93)			
		IMV/D (Events, *n* = 28)		Death (Events, *n* = 16)		IMV/D (Events, *n* = 28)		Death (Events, *n* = 16)	
		HR (95% CI)	*p*	HR (95% CI)	*p*	HR (95% CI)	*p*	HR (95% CI)	*p*
Covariate	Effect								
VitD	<20 ng/mL	5.75 (1.73–19.09)	0.004	11.27 (1.48–85.55)	0.02	3.99 (1.20–13.28)	0.02	7.97 (1.05–60.60)	0.04
Age	≥60 years	4.03 (1.35–12.02)	0.01	9.35 (1.21–72.45)	0.03	3.91 (1.26–12.07)	0.02	9.25 (1.16–73.88)	0.04
Gender	Male	1.63 (0.73–3.61)	0.23	2.31 (0.74–7.19)	0.15	1.38 (0.61–3.11)	0.44	2.04 (0.64–6.48)	0.23
Comorbidity	Any	3.48 (1.36–8.87)	0.009	5.90 (1.31–26.48)	0.02	1.91 (0.72–5.02)	0.19	3.15 (0.68–14.59)	0.14

Abbreviations: CI confidence interval; HR, hazard ratio; IMV/D, invasive mechanical ventilation and/or death; VitD, vitamin D.
